# Gluten Assessment in Beers: Comparison by Different Commercial ELISA Kits and Evaluation of NIR Analysis as a Complementary Technique

**DOI:** 10.3390/foods10061170

**Published:** 2021-05-23

**Authors:** María del Pilar Fernández-Gil, Edurne Simon, Anna Gibert, Jonatan Miranda, Esther Roger Alcoba, Olaia Martínez, Elisenda Vilchez Cerezo, María Ángeles Bustamante

**Affiliations:** 1 Gluten Analysis Laboratory of the University of the Basque Country, Department of Nutrition and Food Science, University of the Basque Country, 01006 Vitoria, Spain; mariadelpilar.fernandez@ehu.eus (M.d.P.F.-G.); edurne.simon@ehu.es (E.S.); olaia.martinez@ehu.eus (O.M.); marian.bustamante@ehu.eus (M.Á.B.); 2 GLUTEN3S Research Group, Department of Nutrition and Food Science, University of the Basque Country, 01006 Vitoria, Spain; 3 Associació Celíacs de Catalunya, Nutrition Department, 08026 Barcelona, Spain; annagibertc@gmail.com (A.G.); eroger@celiacscatalunya.org (E.R.A.); elisenda@celiacscatalunya.org (E.V.C.)

**Keywords:** gluten, beer, NIR, ELISA, brewing, safety

## Abstract

Traditionally, beers are made with gluten-containing cereals. It is crucial to have rapid analytical methodologies that allow gluten content control of the beers for celiac consumers. We assess the content of gluten in 65 conventional and 41 gluten-free labeled beers commercialized in Europe and compare the results in a subgroup of 71 beers with three ELISA kits. This research allows gathering information on the potential complementary utility of NIR analysis applied to gluten analysis of gluten-free beers in terms of time saving. Results obtained with the ELISA technique identified competitive R5 to be the most sensitive in detecting the prolamins, by eliciting a higher number of beers containing gluten above 20 mg/kg. The gluten content in conventional beers tested increased with the presence of wheat as raw material and with the use of ale-type yeasts. By using competitive R5, 3 out of the 41 gluten-free labeled beers appeared to contain gluten above 20 mg/kg, and conversely, 15 out of 65 of the conventional beers showed a gluten content below this threshold. According to our approaches, NIR did not achieve a suitable correlation with ELISA results, neither for gluten quantification nor for discrimination, and therefore, it cannot be proposed as a complementary technique.

## 1. Introduction

Beer represents one of the most frequently consumed beverages worldwide, and brewing is the oldest biotechnological process [1]. In fact, the average annual per capita consumption of beer in North America and Europe reaches 74 and 63 kilograms, respectively, while wine consumption is 8 and 18 kilograms [2]. This fermented alcoholic beverage is derived from malted cereal grains such as barley or wheat, water, hops, and yeast strain [3].

In traditional brewing, the maltose obtained from malted barley is used by yeast during fermentation, resulting in alcohol and carbon dioxide production [4]. Proteins, derived primarily from barley and to a lesser extent from yeast, contribute to the characteristic features of the final product including haze formation, foam retention, foam stability, and flavor [5,6]. Traditional beers (ale, lager, etc.), made mostly from barley or wheat, are manufactured at different fermentation temperatures and yeast types (top, bottom, spontaneous) [3]. In order to meet consumer tastes and needs, new types such as nonalcoholic, fruit-flavored, craft beers, or gluten-free (GF) beers have become more prominent [7]. In fact, the GF beer market has taken off in the last few years [8].

Conventional beers are unsuitable for people with gluten-related diseases such as celiac disease, which affects 1% of the general population, although this issue has been questioned by several authors [9]. During mashing and fermentation, most of the protein fraction is removed by precipitation, lautering processes, and enzymatic hydrolysis [4]. As a result, short peptide fragments and free amino acids remain in the beer. Taking into account that the remaining gluten fraction could maintain its toxicity for celiac individuals [10], further efforts are needed to obtain a beer below the safe threshold established by European Regulation No 828/2014 [11]. According to this regulation, gluten-free foods are those that contain no more than 20 mg/kg of gluten. It also defines very low gluten foods as those that contain wheat, rye, barley, oats, or their hybrid varieties, which have been specifically processed to reduce their gluten content and do not contain more than 100 mg/kg.

The main strategies to reduce the presence of gluten in beers are either based on the use of GF cereals and seeds as raw materials (rice, maize, quinoa) or on enzymatic treatments to process barley-based beers [6]. Enzymes such as prolyl endopeptidase (PEP) (EC 3.4.21.26) are generally added at the beginning of fermentation of GF barley-based beers [9]. Nevertheless, this well-intentioned procedure could also affect some parameters such as foam stability or turbidity [12]. In addition, the lack of stabilization process or filtration in the manufacturing of craft beers entails higher gluten content, compared to industrial ones [13]. It has also been described that lasting large protein fragments in the gluten-reduced beers could be caused by incomplete digestion by those proteolytic enzymes, compelling further optimization of enzymatic use [1]. In fact, celiac disease immunogenic motifs have been found in residual gluten peptides that remained in beer treated with PEP [14].

Gluten is a complex mixture of protein, prolamins, and glutenins present in a wide range of unprocessed and processed matrices. This fact, in addition to the difficulty of achieving an adequate reference material, especially for hordeins [15], leads to some well-known limitations to gluten determination in beers and in general in food. Enzyme-linked immunosorbent assays (ELISA) is the currently accepted method by Codex Alimentarius for the detection of gluten in food products. It is a type I method and hence is the only method for establishing the accepted value of gluten content and the standard specifically indicates the use of the R5 antibody (Mendez Method) [16]. A study by Allred [17], using ELISA methods on celiac sera, has shown that residual peptides of gluten-reduced beers are able to react against serum from a subset of active celiac patients. The usual antibodies used for gluten determination, such as R5 or G12, only detect some of the immunotoxic peptides [18], and therefore, certain authors have proposed liquid chromatography–mass spectrometry (LC–MS) for gluten analysis in hydrolyzed samples as a more reliable technique, compared to ELISA method [1]. Since LC–MS is still in development and requires a substantial investment together with highly qualified staff, alternative methods are being proposed [18]. In line with this, one of the most affordable analytical methods and widely used by the brewing industry is near-infrared spectroscopy (NIR) [19]. NIR proved its capacity to quantify physical properties in food products such as protein or moisture content long ago [20]. Furthermore, it seems that small amounts of nutrients such as fatty acid or vitamins could be determined in food by NIR [21]. Some research studies propose this technique as complementary to conventional methods for gluten level determination, mainly in flours [22,23]. However, no approach has been conducted in the field of beers. This absence becomes more relevant considering that beer can be, in terms of quantity, one of the main contributors to transgression in celiac adult diet [24].

Hence, the aim of this study was to assess the content of gluten in conventional and GF-labeled beers commercialized in Europe by competitive R5 ELISA kit and compare the results in a subgroup of 71 beers with the other two commercially available ELISA kits. Since the NIR technique has complementary proprieties to ELISA, in terms of time and reagent saving, we evaluate the potential correlation between NIR analysis and ELISA results, which may lead to developing a complementary contamination-monitoring sensor for brewing.

## 2. Materials and Methods

### 2.1. Beer Sampling

In total, 160 barley-based beers (labeled as GF and conventional) sold in Spain but with national and international (Belgium, Germany, United Kingdom, the Netherlands, Czech Republic, Italy, Estonia, and Norway) origins were specifically selected from November 2019 to February 2020 to analyze descriptor factors such as manufacturer, yeast style, and wheat inclusion as raw material and original extract. Some of them were also found to include other cereals with gluten (wheat, rye, and oat) or without this protein (rice, millet, and corn) as raw materials. Exclusion criteria for the cohort were as follows: flavored beers, mixed with other alcoholic drinks, and toasted beers. Detailed descriptors of this set of beer are provided in Table 1. From this group, 71 beer samples were used for the comparison of the three ELISA kits used. For the NIR correlation approach, apart from the 106 beer set, another 229 samples were used but without recording information about descriptors.

### 2.2. Gluten Determination by Enzyme-Linked Immunosorbent Assay

Samples were degassed by shaking before the analysis. The gluten content of the overall beer samples was analyzed by commercially available kits: RIDASCREEN Gliadin competitive kit (R7021, R-Biopharm AG, Darmstadt, Germany), based on R5 monoclonal antibody. In the case of the 71 beer cohort, other additional tests were used based on R5 antibody (INGEZIM Gluten Hidrolizado R.30.GLH.K2, Ingenasa, Madrid, Spain) or on the detection of the T-cell stimulatory epitope of α-20 Gliadin (GLUTEN TEC 5171GT(10)03.20, EuroProxima, Arnhem, the Netherlands). All three assays are recommended for hydrolyzed foods and marketed in Spain. RIDASCREEN Gliadin and GLUTEN TEC are competitive enzyme immunoassay (ELISA), while INGEZIM gluten is a direct test. Table 2 provides a description of the manufacturer calibrant, extraction volume of reagents as well as approximate time requirement for each test.

Manufacturers of both competitive kits detailed specific extraction protocols for beers but the direct assays did not. Then, after an internal test (data not shown), the extraction protocol proposed by the manufacturer for tannins or polyphenols was employed in the INGEZIM Gluten Hidrolizado kit.

The samples containing gluten at a concentration greater than the dynamic range of the ELISA kits were diluted according to the manufacturer’s recommendations (minimum dilution to be in range was used). The assays were performed in duplicate for each sample, and results were expressed in parts per million of gluten (mg of gluten/kg of sample). Considering that RIDASCREEN Gliadin competitive and GLUTEN TEC kits measure prolamin content, the results were converted into gluten content by multiplication by a factor of two.

### 2.3. NIR Spectroscopy Analysis

After degassing, the overall set of samples (*n* = 335) were scanned from 1400 to 2400 nm in Zeutec Spectraanalyser 2.0 (double beam NIR) (Germany) filter-spectrometer. The analysis was carried out by every time selecting 7 NI -filters from 19 included (2348 nm, 2336 nm, 2310 nm, 2270 nm, 2230 nm, 2208 nm, 2190 nm, 2139 nm, 2180 nm, 2100 nm, 1982 nm, 1940 nm, 1818 nm, 1778 nm, 1759 nm, 1734 nm, 1722 nm, 1680 nm, and 1445 nm). Each sample was analyzed in duplicate to reduce measuring errors at a temperature of 22 ± 2 °C. Chemometric models of qualitative discrimination and qualitative determination of gluten were prepared from ELISA kits’ data using Application worxG2 software with the multiple linear regression (MLR) model. Due to the big sample number (*n* = 335), scatter correction was performed in the model, not including the outliers proposed by the software. Three types of outlier were described: (1) chemist, (2) spectroscopy, and (3) the sum of both. The first outlier was calculated for each spectrum included in the calibration and points out the samples that exceed three times the normalized Mahalanobis distance. The second outlier was considered for each spectrum included in the correlation process and indicates all those that exceed 2.5 times the typical error of the estimate. The limit for the sum of both outliers was settled at three times. The outlier amount rejected did not reach 10 percent of the total samples. The representativeness of the model was assessed based on the coefficient of determination (R^2^). The reliability of the equipment was confirmed by alcohol grade and protein content of 71 beers set (*R^2^* = 0.84 and *R^2^* = 0.732, respectively).

Qualitative discrimination was performed by dummy variables, as reported elsewhere [22]. Codex Alimentarius categorizing GF beer, very low gluten (LG) beer, and gluten-containing (GC) beer was the criteria for dummy variables created. A set of 18 beers was used for validation of the resulted model from Codex categorizing 6 GF beer, 6 very low gluten beer, and 6 GC beer.

### 2.4. Statistical Analysis

Results were presented as mean and standard deviation. One-way analysis of variance was employed to compare gluten content carried out by three ELISA kits in 71 beers. Tukey post hoc test was used for multiple comparisons among groups. Due to the skewed distribution, the gluten content variable was logarithmically transformed to obtain a more symmetrical distribution. Additionally, to perform a paired sample test, statistical differences of gluten, calculated through different ELISA kits, were analyzed with Wilcoxon test (R5 competitive × R5 direct, R5 competitive × α20gliadin competitive, and R5 direct × α20gliadin competitive).

With regard to the comparison of R5 competitive ELISA data in 71 beers by different classification items, the difference in gluten content and frequencies of GC beers were analyzed by Mann–Whitney U and Pearson’s chi-square tests, respectively. Similarly, Pearson’s chi-square test, followed by a post hoc approach (Bonferroni correction), was used to determine differences in frequencies of Codex classification between commercial ELISA kits. Significant differences were considered at *p* < 0.05. Statistical analysis was performed using IBM SPSS Statistics 24.0 (SPSS Inc., Chicago, IL, USA).

## 3. Results

### 3.1. Gluten Analysis in Beer by Three Different ELISA Kits

The analysis of gluten in 106 beers commercialized in Europe and composed of 41 GF-labeled beers made from gluten-reduced ingredients and 65 conventional beers was carried out by R5 competitive assay (Supplementary Table S1). Moreover, 71 out of the 106 were also measured by R5 direct and α20gliadin competitive kits. See Table 1 for the characterization of the 106 beers under study and Table 3 and Supplementary Table S1 for results of the gluten content of the beers analyzed using the three different kits.

For the conventional beers, significant differences were noted with regard to yeasts style (ale or lager) and wheat inclusion using the R5 competitive. Ale- and wheat-containing beers showed greater gluten content than lager and beers without wheat, respectively (*p* = 0.001 and *p* < 0.001). By contrast, no difference was observed for manufacturer or original extract beer descriptors. The other two ELISA kits also confirmed these significant differences between ale and lager as well as with wheat inclusion or not.

In the case of beers labeled as GF, craft and lager-style beers, as well as beers made without wheat as ingredients showed higher gluten content than industrial and ale-style beers, and beers made with wheat. The fact that beers labeled gluten-free and that included wheat as raw material had lower amounts of gluten probably implies the inclusion of additional steps in the production process and/or the use of enzymes to hydrolyze gluten (e.g., PEP). These differences were, however, not statistically significant in the subset of GF beers.

**Figure 1:**
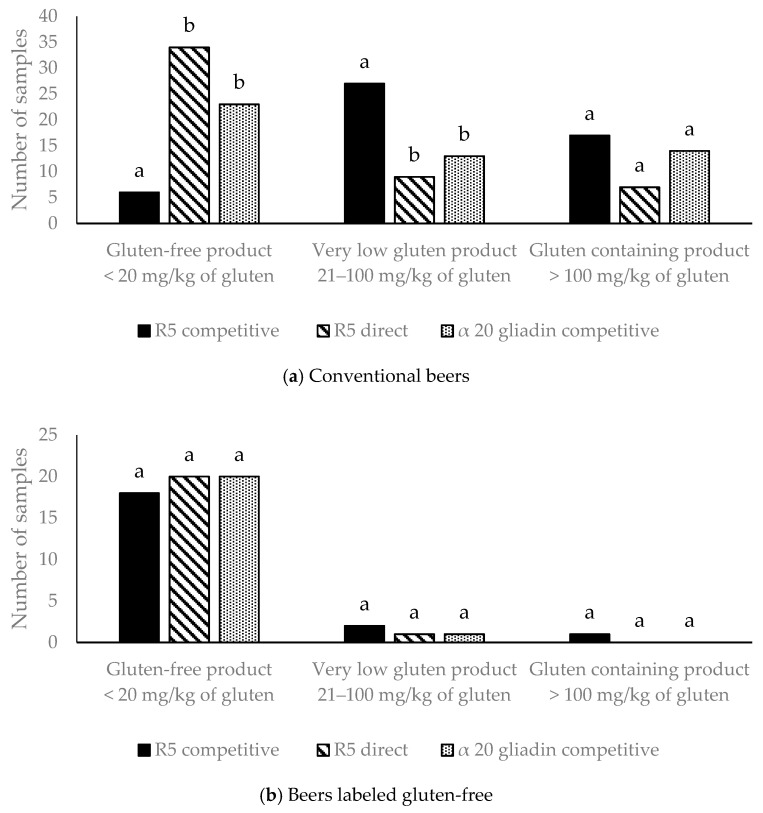
Number of (**a**) conventional beers and (**b**) beers labeled gluten-free from the cohort in each gluten threshold according to Codex standard [16] and European legislation [11] resulting from the analysis performed with three commercial ELISA kits used. Analysis was performed with the chi-square test. Bars with different superscript letters are significantly different (*p* < 0.05).

As far as the conventional beers, 23% of them (15/65) showed a gluten content below 20 mg/kg of gluten by using the R5 competitive assay (Supplementary Table S1). This was a foreseeable outcome, although it would be interesting to ascertain the reason these 15 beers were not intended to be gluten-free showed values within the threshold. Processes such as centrifugation and filtration probably help reduce the presence of gluten, but these processes are not described on the label of all commercial beers. Of interest too is to mention that depending on the ELISA kit used for gluten determination, different results were obtained. Figure 1a describes the number of conventional beers with results in each gluten interval defined by the Codex standard [16] and European legislation [11] (GF product for gluten content below 20 mg/kg, very low gluten product for gluten content between 21 and 100 mg/kg, and gluten-containing product for gluten content over 100 mg/kg).

In the cohort of 71 samples, the number of beers with a gluten content below 20 mg/kg was higher when using the R5 direct (35) or the α20gliadin competitive (23) than when using the competitive R5 (6) (*p* < 0.001). This correlates with the low number of beers containing very low gluten (20–100 mg/kg) when gluten was determined by R5 direct (8) or α20gliadin (13) assays, compared to a higher number when R5 competitive was used (27) (*p* < 0.001). Although the number of beers classified as gluten-containing products by R5 direct was half (7), compared to competitive R5 (17) and α20gliadin (14), the difference did not reach significance.

In the subset of beers labeled as GF, 3 out of 41 samples analyzed by the R5 competitive kit were found above the limit of gluten content (20 mg/kg of gluten) (Supplementary Table S1). The comparison performed by three ELISA kits in 21 of the beers labeled as GF revealed no differences among the kits in each gluten range (Figure 1b). Nevertheless, the result obtained with R5 competitive assay, 3 beers out of 21 samples with more than 20 mg/kg of gluten did not match in all cases with the one resulting from the other two ELISA kits (Figure 1b). When using the other two kits, only one beer showed a level of gluten above 20 mg/kg. From these three beers containing more than 20 mg/kg when using the R5 competitive, none of them had wheat as an ingredient in the label (Supplementary Table S1). Two of them were industrial, and two were lager style. The one with the highest gluten detected (343 mg/kg) was crafted (Supplementary Table S1).

### 3.2. ELISA Results and NIR Analysis Correlation

Table 4 presents correlation statistics between R5 competitive ELISA and NIR analysis for the overall set of beers (*n* = 335). The obtained correlation coefficient (R^2^) for the overall beer sample was 0.167. Correlation statistic improved when overall beer set stratification according to gluten content (0–19 mg/kg, 20–100 mg/kg, and >100 mg/kg) was carried out, showing R^2^ values from 0.139 to 0.592. In none of the cases, correlation reached the 0.7 threshold value to be considered a high correlation.

Two qualitative–discriminatory models were performed. Although similar correlation statistic values were reached for both models, the equation based on a dummy variable with three categories (GF beer, LG beer, and GC beer), showed a slightly higher R^2^ value than that based on GF and non-GF categories (0.201 vs. 0.185).

Specific correlation statistics for the well-characterized beer cohort (*n* = 71) are defined in Table 5. Gluten’s mean content and standard deviation were calculated for the results obtained by three different ELISA assays (R5 competitive, R5 direct, and α20gliadin competitive). The analyzed beer samples presented a high variance of gluten content between them and in a wide range, which resulted in high SD. The correlation between each ELISA kit and NIR analysis was comparable in the three cases. For qualitative–discriminatory models stronger R^2^ values were obtained, with values over the 0.5 threshold to consider moderate correlation (R5 competitive = 0.500, R5 direct = 0.505 and α20gliadin competitive = 0.520).

The cross-validation conducted with a new beer set for the R5 competitive equation showed that, of the 18 beers, 11 samples were misclassified.

## 4. Discussion

From its long background and history of use and its establishment as the Codex Alimentarius type I method for gluten detection, ELISA stood out from the rest of the available methods for gluten detection and quantification [25]. In the case of fermented beverages such as beer, R5 competitive ELISA is currently the only method validated by collaborative studies to quantify gluten [25]. Nevertheless, other ELISA kits have been used for gluten characterization of beers by the scientific community in the past [26] and in the last 10 years [18]. For this reason, in the present research, two other commercialized assays were used, apart from R5 competitive.

The selection of those kits was based on several criteria, one of the most important of which is quality assurance. Beer companies rely on laboratories certified by ISO/IEC 17025 for gluten quantification and determination; in the case of our laboratory, it has been certified for gluten analysis in hydrolyzed matrixes since 2014. To archive this accreditation, it is essential to take part in interlaboratory testing. Food Analysis Performance Assessment Scheme (Fapas^®^) is a worldwide food chemistry proficiency with specific gluten-testing in beer. The 2019 Fapas^®^ report indicates that RIDASCREEN Gliadin competitive was the most widely used ELISA kit for gluten testing in beers (27 out of 36 laboratories). Although less widely used, other ELISA tests such as RIDASCREEN Gliadin, INGEZIM Gluten Hidrolizado, and Hygiena GlutenTox ELISA Competitive G12 (KIT3012) have been employed. RIDASCREEN Gliadin was rejected from our selection because the competitive kit produced by the same manufacturer was a better option for beer analysis [27]. Therefore, RIDASCREEN Gliadin competitive, INGEZIM Gluten Hidrolizado, and Hygiena GlutenTox ELISA Competitive G12 constituted our first option to conduct ELISA comparison. Two kits are based on R5 antibody, as proposed by Codex [16], and one is based on G12 antibody, as an alternative to R5. However, due to a lack of quality assurance of GlutenTox ELISA Competitive G12 declared by the manufacturer, this kit was not finally included in the selection. In substitution, as an alternative to R5, a GLUTEN TEC kit was used, based on α-20 Gliadin [28].

Our data showed distinct mean values of gluten depending on the ELISA assay used. In the analyzed beer sample set, R5 competitive ELISA (RIDASCREEN Gliadin competitive) detected more amount of gluten than α20gliadin competitive (GLUTEN TEC) and R5 direct (INGEZIM Gluten Hidrolizado), respectively. R5 competitive kit is, taking into account this comparison, the most restrictive method, and therefore, deviations from the use of the R5 competitive ELISA method may derive into a safety risk to the celiac consumer. Accordingly, specific research about gluten fragment detection indicated that R5 competitive ELISA provided higher prolamin concentration in barley-based beer than the noncompetitive format of the same manufacturer [27]. Similar inferences were made in 51 commercial Belgian barley malt beers, measured by the same kits. R5 competitive ELISA identified less than half the number of GF beer than did direct assay (18 vs. 45) [29]. However, in the present approach, the noncompetitive kit comes from a different manufacturer and is a direct ELISA and thus not based on sandwich methodology.

Highly variable proteolytic peptides resulting from the brewing processes lead to more appropriate recognition than the requirement of only one epitope as competitive or direct ELISA tests do. A recent review showed that depending on the antibody, contradictory results can be obtained using the same technique (competitive ELISA) for gluten detection of gluten in the same beer samples [18]. In our case, both competitive kits (R5 and α20 gliadin) provided significantly different results both in the number of beers with a gluten content below 20 mg/kg and gluten mean values. R5 antibody provided an optimal reproducibility but required the use of a conversion factor from the recognized peptide to protein (gluten), which is not always suitable [30]. However, α20 gliadin competitive results were in the same order of magnitude as those reported by Mujico et al. in the interlaboratory study for commercial beer (179 µg/kg) [31]. By contrast, the proportion of beer that we found below 20 mg gluten per Kg (23/71) is quite similar to other research conducted with thirty Belgium beer by G12 competitive ELISA kit (8/30) [10].

Since the R5 competitive ELISA was the most restrictive test employed, an in-depth analysis of detected gluten was conducted for 71 beer set by different classification items. Our results in conventional beers reveal greater gluten content in ale than lager beers and in the ones including wheat than those that do not have this cereal as an ingredient. Other researchers have also reported gluten content changes according to beer types. In 2005, Kanerva et al. found remarkable differences between lager and wheat beers using the Tepnel Biokits ELISA assay, based on mAb 401.21 antibody, also called Skerrit antibody [26]. In fact, the prolamin content of wheat beer was about one thousand times that of lager beer (6 vs. 6000 mg/kg). The reason behind wheat-based beers containing a greater amount of gluten could be due to the percentage of prolamins in wheat is higher than in barley (32.6% vs. 25%) [32]. Another reason could be related to the brewing process. The addition of malted cereal or not, the use of clarification processes, such as filtration, centrifugation, or a cold-break step, can influence the gluten content in beer [29]. The malting process implies germination of the grain that provides the necessary proteases to degrade storage proteins such as hordeins [9]. Many of the conventional wheat beers present higher turbidity; therefore, it can be expected that beer clarification processes are not used, at least in conventional wheat beers. The influence of brewing yeast on gluten content in the final product was previously pointed out by Tanner et al. in 2013 using a sandwich noncompetitive assay based on Skerrit antibody; they described a mean hordein (gluten) values of 62.7 and 3120 mg/kg for lager and ale beers, respectively [33]. More recently, in 2017, R5 antibody data extracted from a multiplex competitive ELISA for the detection and characterization of gluten in 46 beers did not show any difference between ale and lager beer types (mean value 88 vs. 112 µg of gluten per mL of beer). However, the data obtained from multiplex assay indicated the higher gluten content of wheat beers in comparison with barley (mean value 2644 vs. 100 µg of gluten per mL) [34]. Similar results were found in 51 Belgian beers measured by R5 competitive ELISA. They analyzed 13 beer types and highlighted that in wheat and gueuze beer, gluten content was extensively most abundant [29]. Considering that ale yeast is used for gueuze beer production, results from the present research are completely in agreement with those reported with Belgian beers. Indeed, no correlation exists between the original extract of barley beers and gluten content [29], which we confirmed. As a whole, and supported by the literature, both the main ingredients as well as the yeasts used are relevant contributors to the gluten content of beers.

Regarding beers labeled as GF, no significant quantitative disparities among beer types were found. Notwithstanding, it is important to highlight that, according to the R5 competitive assay, 3 out of 41 beers labeled as GF (one India Pale Lager beer, one German Pils beer, and one Dry Hopped Ipa beer) contained more than 30 mg/kg of gluten, and consequently, they were over the threshold stated in the legislation [11]. These apparently unexpected results were previously reported by Van Landschoot [35]. After analyzing 45 Belgian beers, they indicated that gluten content for GF beers was in the range of 5–8 mg/kg by using the R5 sandwich test. However, R5 competitive ELISA data suggested that not all of these GF beers should be considered GF. From our study, it is worth noting the case of craft beer labeled gluten-free, which had a gluten content of over 300 mg/kg.

The use of the PEP enzyme for GF brewing is widespread, both in industrial and craft breweries [6]. Large brewing companies have developed standardized processes and steps, such as centrifugation and filtration, that successfully render beer GF. By contrast, craft brewers can only approximate this goal due to lower standardization. The human factor prevails over the mechanical one in manufacturing to obtain individualized final outcomes. This fact could justify the results presented here.

Another worthwhile outcome of the present research was the proportion (15 out of 65) of conventional beer that contained gluten levels below 20 mg/kg of gluten. Further research would be needed to understand the factors that have yielded to those beers so that this could be consistently applied to all batches. In other studies, commercial beer analyzed by R5 competitive [36], Skerrit sandwich [33] or G12 competitive ELISA kits [10], also determined less than 20 mg/kg of gluten; 28%, 50%, and 27%, respectively (beer proportion was of 7 out of 25, 25 out of 50 and 8 out of 30). In their research, Watson et al. shed light on the divergences among studies [29]. In their gluten content quantification by R5 competitive ELISA kit, they described that 10 out of 51 beers did not contain more than 20 mg/kg of gluten in any of the three batches analyzed. However, in 13 other beers, batch-to-batch variation was observed, none of the batches being below the guideline threshold. Overall, our outcomes in GF-labeled and conventional beers reinforce the importance of the brewery companies and the brewery technology used in the gluten content of the final product.

Nowadays, Codex Alimentarius establishes the methodology ELISA R5 for the determination of gluten in foods as a type I method. Fermentation and hydrolysis processes, so common in food preparation, can break down gluten, making it difficult to determine. Thereby, in fermented and hydrolyzed foods, the use of competitive [16] or similar ELISA is compulsory, at least in those foods commercialized in Europe [37]. However, as Panda and Garber extensively described, gluten detection and quantification in beers by ELISA analysis requires the use of adequate reference material, reagents, and analysis times [18].

The time required to perform routine quality control testing of raw material, intermediates, and final products slows down and makes brewing processes more expensive. These ought to obtain analytical data to ensure the suitability of steps performed (enzyme treatment, filtration and decantation processes, etc.) [19]. In GF brewing, obtaining quicker reliable results about gluten content could remove additional steps during brewing or longer-acting times of the enzymes. In addition, it would allow optimization of analysis times and perform verification analyzes only on those samples or batches that require it, or when it is necessary to confirm the screening results by accredited methods.

NIR has been used successfully for the determination of numerous analytical parameters (protein, moisture, hardness, water abortion, fat, starch, sugar fiber) in cereals and cereal products, milk and dairy products, meat, fish, fruits and vegetables, etc. [20]. A clear example of its value in the field of grains is its establishment as the official method of the US Federal Grain Inspection service for determining the amount of protein in wheat [38]. Several researchers have also described NIR applicability for gluten analysis in cereal and cereal-based foodstuffs. In fact, NIR was used for identification or quantification of gluten in wheat grain [22] and for cereal-based food matrices that contain this protein naturally [39], as well as in flours, batter, or cookies in which this was determined in order to guarantee its absence [23,40].

NIR has been employed in beers for the determination of other parameters such as pH, alcoholic degree, bitterness, primitive dry extract, and total soluble nitrogen [19,41]. However, to our knowledge, no scientific research provides evidence about its usefulness for gluten analysis in this alcoholic beverage. It is important to highlight that the NIR technique has several advantages, such as speed in obtaining analytical results, the low cost per analysis, little or no sample preparation, and the lack of need for additional chemical reagents. Considering that those characteristics could solve some of the limitations of ELISA gluten analysis during the brewing process, we postulate the potential use of NIR as a complementary analysis applied to gluten determination during the manufacturing of gluten-free beers. It could be useful for screening a greater number of production batches than ELISA, for instance, which is a crucial aspect, as pointed out before. However, since NIR is an indirect method based on ELISA results, a confirmatory ELISA test would always be necessary.

ELISA tests for gluten quantification use a considerable volume of chemical reagents such as fish gelatin, polyvinylpyrrolidone, extraction buffer, or ethanol (at different grades). Additionally, the completion of the assay takes about 75 to 120 min. By contrast, NIR analysis of beer does not entail any chemical reagent and the complete assay for each sample lasts 30 s. In order to answer our hypothesis, we used a filter spectrometer (1200–2600 nm), followed by MLR analysis. This equipment was selected for two main reasons. Firstly, these instruments are simpler and cheaper than monochromators and diode array spectrometers [20]. Regarding the feasibility of the technique, these are relevant features for small-to-medium breweries. Secondly, the use of the partial least squares method (PLS) was avoided in the correlation. As stated by Wesley et al., PLS removes spectral regions of protein content that have a negative impact on the determination of gliadin and glutenin content [39].

Our first approach for NIR analysis was carried out with 335 beers available on the Spanish market. It is essential to clarify whether a potential correlation occurs between R5 competitive ELISA and NIR results in a large number of beers. The correlation statistics did not achieve the quality goals (*R^2^* > 0.7), neither when beers were evaluated as a whole nor when samples were stratified by the regulation (EU) No 828/2014 [11]. Specifically, the law sets levels of gluten for foods claiming to be either “gluten-free” (less than 20 mg per 1 Kg food) or “very low gluten” (less than 100 mg per 1 Kg food). Beers containing more than 100 mg/kg of gluten were identified as GC beer in this first approach. The overall correlation coefficient obtained for the beer sample (R^2^ value of 0.167) was a long away from the raw data results described by others measuring wet gluten content (*R^2^* value of 0.53) and gliadin content (*R^2^* value of 0.46) in whole-wheat grain and flours, respectively [39,42]. Nevertheless, after the PLS application, the calibration statistics reach acceptable values in previously mentioned research [39,42].

Further analysis of our results showed that correlation statistics improved with increasing levels of gluten concentration (from an R^2^ value of 0.139 for GF beers to an R^2^ value of 0.592 for samples with more than 100 mg/kg). In agreement with these results, Albanell et al. reported a convenient NIR calibration for wet gluten content in flour and batter with the exception of concentrations between 0 and 100 mg per 1 Kg food [23]. Therefore, they concluded that this method was not suitable for determining GF products. Along these lines, it must be highlighted that other studies with high correlations for wet gluten or gliadin quantification by NIR were carried out with high gliadin-content flour (3.55–7.55%) [39] and high wet-gluten wheat grain (31.7%) [42].

As an alternative approach for gliadin or gluten quantification, a remarkable trial was conducted by Garcia-Molina et al. using NIR [22]. They confirmed the effective identification of low-gliadin wheat lines by NIR, classifying samples by binary code in a dummy variable. Mirroring this procedure, we generated two dummy variables for sample discrimination: (a) according to Codex categorizing (GF, LG, GC) and (b) dichotomic categorizing in GF and non-GF products. Unfortunately, the correlation parameters presented here for discriminatory models by NIR analysis indicated that this methodology was not suitable for discrimination purposes. Different foodstuff (whole grain and flour vs. beer) and gluten content (minimum and maximum gluten content of samples were 3057–181986 vs. 3.4–3717) can justify the discrepancy between Garcia-Molina et al. research and the ongoing one [22].

According to the results, it seems that NIR analysis was not suitable for gluten quantification or GF sample discrimination in beers. However, to be sure that this affirmation was not related to the ELISA kit used for the correlation, we performed a second approach for NIR correlation with 71 beers using 3 different ELISA kits proposed for gluten analysis in beers. Results demonstrated that the lack of correlation between NIR and ELISA was independent of the ELISA used (R5 competitive, R5 direct, and α20gliadin competitive). In fact, similar correlation values were obtained for the evaluation of gluten content as well as GF product discrimination among the three ELISA kits. As with the 335 beer group, the best correlation, defined as R^2^, was reached for Codex-categorizing discrimination. For this reason, cross-validation was conducted with a set of 18 beers for *Codex*- categorizing discrimination according to the R5 competitive ELISA kit calibration model. The classification error percentage reached 61%, confirming that the model proposed was not completely reliable.

## 5. Conclusions

The lack of a green technique and affordable monitoring of cross-contamination during the brewing process adds to the financial handicap of inconsistent GF labeling of beers. In this study, research presented for the first time shows the potential use of NIR as a complementary technique to ELISA in gluten determination of commercial beers. Our result does not allow us to put forward NIR analysis as a sensor for gluten quantification or GF sample discrimination in beers. For the moment, it is not possible for breweries to use NIR analysis to widen the control throughout the brewing process. However, this work can be considered as a first milestone in the field where future improvement of NIR technique or new chemometric models leads to better alternatives in the gluten analysis.

The study also leads us to conclude that the R5 competitive ELISA kit is the most sensitive of the three immunologic methods used here to discriminate low levels of gluten in beer. The analysis performed on conventional beers highlights the significance of lager yeast selection or wheat exclusion on the brewery process to reduce the gluten content of the final product. As for the results on the selected labeled GF beers, we detected some outliers of gluten content that would require attention from the regulatory bodies. On the contrary, some of the conventional beers, not specifically manufactured to be GF, showed levels of gluten below 20 mg/kg. These results are, in all likelihood, pointing at the need for more research in the field of fermented and hydrolyzed foods to understand better all the factors that can yield to produce consistent batches of GF beer within the regulatory threshold, in addition to highlighting the importance of making use of approved validated methods to determine its gluten content with the final aim of protecting celiac consumers.

## Figures and Tables

**Table 1:** Characteristic of beer sets.

Beer Descriptor		Sample Number Labelled GF	Sample Number Conventional
Manufacturer			
	Craft	16	21
	Industrial	25	44
Yeast style			
	Ale	23	37
	Lager	18	28
Ingredients *			
	Made from gluten-containing cereals only	25	53
	Made from gluten-containing and GF cereals	13	11
	Include wheat	3	20
	Without wheat	36	43
Original extract (OE) **			
	Traditional beer (<13 grams of OE per 100 grams of wort)	20	41
	Special beer (13–15 grams of OE per 100 grams of wort)	10	14
	Extra-special beer (>15 grams of OE per 100 grams of wort)	4	8
Country of origin			
	Spain	19	45
	United Kingdom	4	0
	Italy	1	0
	Germany	2	8
	Belgium	11	4
	Norway	1	0
	Netherlands	0	4
	France	1	0
	Check Republic Estonia	1 1	4 0
Overall beer		41	65

GF: gluten free. * Ingredients classification was not stated for four beers. ** Original extract classification was not stated for nine beers.

**Table 2:** Manufacturer’s specified properties of gluten/gliadin kits.

ELISA Kit	Calibrant	Extraction Volume	Approximate Time Requirement		
(Reference)		(10 samples)	Sample Preparation (10 samples)	Implementation (10 samples)	Total Time 10 samples
RIDASCREEN Gliadin Competitive (R7021)	Hydrolysate prolamin from mixture of wheat (PGW gliadin), rye, and barley.	60%ethanol + 10% fish gelatin (90 mL)	30 min	45 min	75 min
INGEZIM Gluten Hidrolizado (R.30.GLH.K2)	Gliadin European standard *	Polyvinylpyrrolidone (1 g) Extraction buffer (25 mL) 80% ethanol (75 mL)	25 min	215 min	240 min
GLUTEN TEC (5171GT(10)03.20)	α-20 gliadin peptide	60%ethanol (45 mL)	25 min	80 min	105 min

PGW: Prolamin working group. * The manufacturer does not provide more detailed information.

**Table 3:** Comparison of the three enzyme-linked immunosorbent assays gluten results in beer´s cohort by different classification items.

Gluten Content mg/kg	Manufacturer			Yeast Style			Include Wheat			Original Extract *		
	Industrial	Craft	*p*	Ale	Lager	*p*	Yes	No	*p*	Beer	Special/extra	*p*
R5 competitive												
Labeled GF												
Mean ± SD	9.723 ± 16.50	47.17 ± 119.6	0.597	8.900 ± 16.01	44.11 ± 112.4	0.678	3.400 ± 0.001	27.42 ± 80.01	0.379	32.58 ± 97.80	7.867 ± 10.94	1.000
Conventional											
Mean ± SD	502.5 ± 972.0	148.4 ± 338.4	0.832	650.1 ± 1064	73.11 ± 134.4	0.001	1145 ± 1206	44.11 ± 30.64	<0.001	549.4 ± 1032	153.3 ± 270.1	0.771
R5 direct Labeled GF												
											
Mean ± SD	1.073 ± 2.615	6.300 ± 14.82	0.526	1.542 ± 3.102	5.095 ± 14.07	0.756	0.250 ± 0.001	3.533 ± 10.08	0.379	4.283 ± 12.19	0.458 ± 0.510	0.885
Conventional											
Mean ± SD	70.29 ± 129.6	22.76 ± 41.67	0.649	89.15 ± 140.6	13.89 ± 27.23	0.001	158.4 ± 157.2	7.872 ± 8.045	<0.001	76.32 ± 138.3	22.22 ± 26.95	0.838
α20gliadin competitive												
Labeled GF												
Mean ± SD	2.415 ± 1.982	11.95 ± 27.65	0.698	2.133 ± 1.787	11.27 ± 25.94	0.324	1.400 ± 0.001	6.822 ± 18.43	0.313	8.433 ± 22.66	3.067 ± 2.594	0.750
Conventional											
Mean ± SD	329.5 ± 554.0	108.1 ± 308.7	0.403	425.4 ± 615.0	56.58 ± 137.1	0.001	784.0 ± 626.8	18.00 ± 15.12	<0.001	357.0 ± 601.4	116.6 ± 209.9	0.804

GF: gluten free; SD: standard deviation * original extract classification was no stated for five beers. Analysis was performed with Mann–Whitney U test.

**Table 4:** Correlation statistics of gluten content or gluten-containing products discrimination of beer determined by R5 competitive enzyme-linked immunosorbent assay and near-infrared analysis in 335 beers.

Gluten Range (mg/kg)	Sample Number	R^2^	Outlier Sample Number
0–19	214	0.139	23
20–100	87	0.184	8
>100	34	0.592	3
0->100	335	0.167	21
Codex categorizing * (GF, LG, GC)	335	0.201	31
Dichotomic categorizing (GF, non-GF)	335	0.185	27

GF: gluten-free (<20 mg/kg of gluten); LG: very low gluten (20–100 mg/kg of gluten); GC: gluten containing (>100 mg/kg of gluten).* Gluten categorizing according to Codex standard [16].

**Table 5:** Evaluation of gluten content or gluten-containing products discrimination of beer cohort by three different enzyme-linked immunosorbent assay and correlation statistics of each one by near-infrared analysis.

		R5 Competitive		R5 Direct		α20gliadin Competitive		
	Evaluated Sample Number	NIR R*^2^*	ELISA Gluten Mean ± SD	NIR R*^2^*	ELISA Gluten Mean ± SD	NIR R*^2^*	ELISA Gluten Mean ± SD	ELISA ANOVA
No categorizing	71	0.435	286.1 ^a^ ± 729.0	0.450	40.37 ^b^ ± 97.30	0.482	187.1 ^a^ ± 435.3	<0.001
*Codex* categorizing (GF, LG, GC)	71	0.500		0.505		0.520		
Dichotomic categorizing (GF, non-GF)	71	0.341		0.472		0.450		

ANOVA, analysis of variance; ELISA: enzyme-linked immunosorbent assay, GF: gluten-free beer; GC: gluten-containing beer; LG: very low gluten beer; NIR near-infrared spectroscopy; SD: standard deviation. Means with different superscript letters are significantly different (*p* < 0.05).

## Data Availability

The data presented in this study are available on request from the corresponding author. The data are not publicly available due to they are property of the GLUTEN3S Research Group and SMAP—Celìacs Catalunya.

## Supplementary Materials

The following is available online at https://www.mdpi.com/article/10.3390/foods10061170/s1, Table S1: Assigned gluten results in commercial beers by three enzyme-linked immunosorbent assays.

## References

[1] Colgrave M.L., Byrne K., Howitt C.A. (2017). Liquid Chromatography-Mass Spectrometry Analysis Reveals Hydrolyzed Gluten in Beers Crafted To Remove Gluten. J. Agric. Food Chem.

[2] FAO FAOSTAT. http://www.fao.org/faostat/en/#home.

[3] Bustamante M., Simón E., Arranz E., Fernández-Bañares F., Rosell C., Rodrigo L., Peña A. (2015). Gluten-Free Spirits and Drinks. Advances in the Understanding of Gluten Related Pathology and the Evolution of Gluten-Free Foods.

[4] Kok Y.J., Ye L., Muller J., Ow D.S., Bi X. (2019). Brewing with malted barley or raw barley: What makes the difference in the processes?. Appl. Microbiol. Biotechnol..

[5] Humia B.V., Santos K.S., Barbosa A.M., Sawata M., Mendonça M.D.C., Padilha F.F. (2019). Beer Molecules and Its Sensory and Biological Properties: A Review. Molecules.

[6] Cela N., Condelli N., Caruso M.C., Perretti G., Di Cairano M., Tolve R., Galgano F. (2020). Gluten-Free Brewing: Issues and Perspectives. Fermentation.

[7] Donadini G., Bertuzzi T., Kordialik-Bogacka E., Cywińska D., Rossi F., Spigno G., Porretta S. (2020). Investigating patterns of millennials’ interest in gluten-free beer in Poland: A question of beer price and alcohol content. J. Food Sci..

[8] Watson H.G., Vanderputten D., Van Landschoot A., Decloedt A.I. (2019). Applicability of different brewhouse technologies and gluten-minimization treatments for the production of gluten-free (barley) malt beers: Pilot- to industrial-scale. J. Food Eng..

[9] Hager A.-S., Taylor J.P., Waters D.M., Arendt E.K. (2014). Gluten free beer—A review. Trends Food Sci. Technol..

[10] Comino I., Real A., Moreno M.e.L., Montes R., Cebolla A., Sousa C. (2013). Immunological determination of gliadin 33-mer equivalent peptides in beers as a specific and practical analytical method to assess safety for celiac patients. J. Sci. Food Agric..

[11] European Commission, (EU) (2014). Commission Implementing Regulation (EU) No 828/2014 of 30 July 2014: Requirements for the provision of information to consumers on the absence or reduced presence of gluten in food. Off. J. Eur. Union L228.

[12] Guerdrum L.J., Bamforth C.W. (2012). Prolamin Levels through Brewing and the Impact of Prolyl Endoproteinase. J. Am. Soc. Brew. Chem..

[13] Fanari M., Porcu M., Zinellu M., Farina D., Scognamillo S., Forteschi M., Luca P. (2017). A preliminary study about gluten levels in Sardinian craft beers. J. Microbiol. Biotechnol. Food Sci..

[14] Watson H.G., Decloedt A.I., Hemeryck L.Y., Van Landschoot A., Prenni J. (2021). Peptidomics of an industrial gluten-free barley malt beer and its non-gluten-free counterpart: Characterisation and immunogenicity. Food Chem..

[15] Tanner G.J., Blundell M.J., Colgrave M.L., Howitt C.A. (2013). Quantification of Hordeins by ELISA: The correct standard makes a magnitude of difference. PLoS ONE.

[16] Alimentarius C. (2008). Revised version of Standard for Foods for Special Dietary Use for Persons Intolerant to Gluten. CODEX STAN 118-2008.

[17] Allred L.K., Lesko K., McKiernan D., Kupper C., Guandalini S. (2017). The Celiac Patient Antibody Response to Conventional and Gluten-Removed Beer. J. AOAC Int..

[18] Panda R., Garber E.A.E. (2019). Detection and Quantitation of Gluten in Fermented-Hydrolyzed Foods by Antibody-Based Methods: Challenges, Progress, and a Potential Path Forward. Front. Nutr..

[19] Sileoni V., Marconi O., Perretti G. (2015). Near-infrared Spectroscopy in the Brewing Industry. Crit. Rev. Food Sci. Nutr..

[20] Osborne B.G., Meyers R.A., McGorrin R.J. (2006). Near-Infrared Spectroscopy in Food Analysis. Encyclopedia of Analytical Chemistry.

[21] Revilla I., Escuredo O., González-Martín M.I., Palacios C. (2017). Fatty acids and fat-soluble vitamins in ewe’s milk predicted by near infrared reflectance spectroscopy. Determination of seasonality. Food Chem..

[22] García-Molina M.D., García-Olmo J., Barro F. (2016). Effective Identification of Low-Gliadin Wheat Lines by Near Infrared Spectroscopy (NIRS): Implications for the Development and Analysis of Foodstuffs Suitable for Celiac Patients. PLoS ONE.

[23] Albanell E., Miñarro B., Carrasco N. (2012). Detection of low-level gluten content in flour and batter by near infrared reflectance spectroscopy (NIRS). J. Cereal Sci..

[24] Elli L., Bascuñán K., di Lernia L., Bardella M.T., Doneda L., Soldati L., Orlando S., Ferretti F., Lombardo V., Barigelletti G. (2020). Safety of occasional ingestion of gluten in patients with celiac disease: A real-life study. BMC Med..

[25] Don C., Halbmayr-Jech E., Rogers A., Koehler P. (2014). AACCI Approved Methods Technical Committee Report: Collaborative Study on the Immunochemical Quantitation of Intact Gluten in Rice Flour and Rice-Based Products Using G12 Sandwich ELISA. Cereal Foods World.

[26] Kanerva P., Sontag-Strohm T., Lehtonen P. (2005). Determination of Prolamins in Beers by ELISA and SDS-PAGE. J. Inst. Brew..

[27] Haas-Lauterbach S., Immer U., Richter M., Oehler E. (2012). Gluten Fragment Detection with a Competitive ELISA. J. AOAC Int..

[28] Mitea C., Kooy-Winkelaar Y., van Veelen P., de Ru A., Drijfhout J.W., Koning F., Dekking L. (2008). Fine specificity of monoclonal antibodies against celiac disease-inducing peptides in the gluteome. Am. J. Clin. Nutr..

[29] Watson H.G., Decloedt A.I., Vanderputten D., Van Landschoot A. (2018). Variation in gluten protein and peptide concentrations in Belgian barley malt beers. J. Inst. Brew..

[30] Scherf K.A., Poms R.E. (2016). Recent developments in analytical methods for tracing gluten. J. Cereal Sci..

[31] Mujico J.R., Dekking L., Kooy-Winkelaar Y., Verheijen R., van Wichen P., Streppel L., Sajic N., Drijfhout J.W., Koning F. (2012). Validation of a new enzyme-linked immunosorbent assay to detect the triggering proteins and peptides for celiac disease: Interlaboratory study. J. AOAC Int..

[32] Belitz H.-D., Grosch W., Schieberle P. (2009). Food Chemistry.

[33] Tanner G.J., Colgrave M.L., Blundell M.J., Goswami H.P., Howitt C.A. (2013). Measuring hordein (gluten) in beer--a comparison of ELISA and mass spectrometry. PLoS ONE.

[34] Panda R., Boyer M., Garber E.A.E. (2017). A multiplex competitive ELISA for the detection and characterization of gluten in fermented-hydrolyzed foods. Anal. Bioanal. Chem..

[35] Van Landschoot A. (2011). Gluten-free barley malt beers. Cerevisia.

[36] Guerdrum L.J., Bamforth C.W. (2011). Levels of gliadin in commercial beers. Food Chem..

[37] Scherf K.A., Catassi C., Chirdo F.G., Ciclitira P.J., Feighery C.F., Gianfrani C., Koning F., Lundin K.E.A., Masci S., Schuppan D. (2020). Statement of the Prolamin Working Group on the Determination of Gluten in Fermented Foods Containing Partially Hydrolyzed Gluten. Front. Nutr..

[38] Department of Agriculture, U.S (USDA) Agricultural Marketing Service. https://www.ams.usda.gov/services/fgis/standardization/wheat-protein.

[39] Wesley I.J., Larroque O., Osborne B.G., Azudin N., Allen H., Skerritt J.H. (2001). Measurement of Gliadin and Glutenin Content of Flour by NIR Spectroscopy. J. Cereal Sci..

[40] Wimonsiri L., Ritthiruangdej P., Kasemsumran S., Therdthai N., Chanput W., Ozaki Y. (2017). Rapid analysis of chemical composition in intact and milled rice cookies using near infrared spectroscopy. J. Near Infrared Spectrosc..

[41] Giovenzana V., Beghi R., Guidetti R. (2014). Rapid evaluation of craft beer quality during fermentation process by vis/NIR spectroscopy. J. Food Eng..

[42] Cozzolino D., Delucchi I., Kholi M., Vázquez D. (2006). Uso de la espectroscopía de reflectancia en el infrarrojo cercano para evaluar características de calidad en trigo. Agric. Técnica.

