# Supplementary material for: Gluten Assessment in Beers: Comparison by Different Commercial ELISA Kits and Evaluation of NIR Analysis as a Complementary Technique
Source: Foods. 2021 May 23;10(6):1170. doi: 10.3390/foods10061170 (PMC8224778; doi:10.3390/foods10061170)
Supplement: Supplementary file 1 [file foods-10-01170-s001.zip › foods-1208772-supplementary.pdf]

Table S1. Assigned gluten results in commercial beers by three enzyme-Linked ImmunoSorbent Assays

| Beer ID | Manufacturer | Yeast style | Ingredients | Gluten content mg/kg | | |
|---|---|---|---|---|---|---|
| | | | | R5 competitive | R5 direct | α20gliadin competitive |
| Labelled GF _1 | Industrial | Lager | Water, barley malt, corn, barley, hops | <10.0 | <0.25 | 6.0 |
| Labelled GF _2 | Industrial | Lager | Natural mineral water, barley malt, hops | <10.0 | <0.25 | <3.6 |
| Labelled GF _3 | Industrial | Lager | Water, barley malt and hops | <10.0 | <0.25 | <3.6 |
| Labelled GF _4 | Industrial | Lager | Water, barley malt, oat, yuzu pasta, shichimi toragashi (sesame), hops and yeast. | 30.2 | 1.5 | 6.8 |
| Labelled GF _5 | Craft | Ale | Water, barley malt, hops, yeast | <10.0 | <0.25 | <3.6 |
| Labelled GF _6 | Craft | Ale | Water, barley malt, hops and yeast | <10.0 | <0.25 | <3.6 |
| Labelled GF _7 | Industrial | Lager | Water, barley malt, corn, hops | <10.0 | <0.25 | <3.6 |
| Labelled GF _8 | Industrial | Lager | Water, barley malt, barley, corn, hops, hop extract | <10.0 | <0.25 | <3.6 |
| Labelled GF _9 | Craft | Lager | Barley malt, yeast, hops | <10.0 | <0.25 | <3.6 |
| Labelled GF _10 | Industrial | Ale | Water, barley malt, hops and yeast | <10.0 | <0.25 | <3.6 |
| Labelled GF _11 | Craft | Lager | Water, barley malt, oatmeal, hops, yeast | 343 | 42.6 | 80.2 |
| Labelled GF _12 | Craft | Ale | Water, barley malt, hops, yeast | 14 | 6.3 | 7.0 |
| Labelled GF _13 | Craft | Ale | Water, barley malt, hops, yeast | <10.0 | <0.25 | <3.6 |
| Labelled GF _14 | Industrial | Ale | Water, barley malt, oat malt, rice flakes, hops, spices, lactic acid, inverted cane sugar, yeast, ascorbic acid | <10.0 | <0.25 | <3.6 |
| Labelled GF _15 | Craft | Lager | Water, barley malt, hops | <10.0 | <0.25 | <3.6 |
| Labelled GF _16 | Industrial | Ale | Barley, wheat | <10.0 | <0.25 | <3.6 |
| Labelled GF _17 | Industrial | Ale | Water, barley, oatmeal, hops, yeast | 58.8 | 9.7 | 4.6 |
| Labelled GF _18 | Craft | Ale | Water, barley malts, oat flakes, hops, yeast | <10.0 | <0.25 | <3.6 |
| Labelled GF _19 | Industrial | Ale | Barley, oatmeal, wheat, water, yeast and hops | <10.0 | <0.25 | <3.6 |
| Labelled GF _20 | Industrial | Ale | Water, barley/wheat malt, hops, yeast, spices | <10.0 | <0.25 | <3.6 |
| Labelled GF _21 | Industrial | Ale | Water, barley malt, hops, yeast, spices | <10.0 | <0.25 | <3.6 |
| Labelled GF _22 | Industrial | Lager | Water, barley malt, rice, hops | <10.0 | | |
| Labelled GF _23 | Industrial | Lager | Water, barley malt, corn and hops | <10.0 | | |
| Labelled GF _24 | Industrial | Lager | Water, barley malt, corn, hops | <10.0 | | |
| Labelled GF _25 | Industrial | Lager | Water, barley malt, corn and hops | <10.0 | | |
| Labelled GF _26 | Industrial | Lager | Water, barley malt, rice, maiz, hops | <10.0 | | |
| Labelled GF _27 | Industrial | Ale | Water, malta, levadura, $CO_2$ | <10.0 | | |

| Beer ID | Manufacturer | Yeast style | Ingredients | Gluten content mg/kg | | |
|---|---|---|---|---|---|---|
| | | | | R5 competitive | R5 direct | α20gliadin competitive |
| Labelled GF _28 | Industrial | Ale | Barley, oat | <10.0 | | |
| Labelled GF _29 | Industrial | Lager | Water, barley malt, hop extract, hops | <10.0 | | |
| Labelled GF _30 | Craft | Ale | Water, barley malt, hops and yeast | <10.0 | | |
| Labelled GF _31 | Craft | Ale | Barley malt, millet, buckwheat, hops, yeast | <10.0 | | |
| Labelled GF _32 | Industrial | Lager | Water, barley malt, rice, yeast and hops | <10.0 | | |
| Labelled GF _33 | Industrial | Ale | Water, barley malt, rice, buckwheat, yeast, hops and spices | <10.0 | | |
| Labelled GF _34 | Craft | Lager | Water, barley and rye malts, hops and yeast | <10.0 | | |
| Labelled GF _35 | Craft | Ale | Water, barley malt, hops and yeast | <10.0 | | |
| Labelled GF _36 | Craft | Ale | Water, malts, hops and yeast | 11.8 | | |
| Labelled GF _37 | Craft | Ale | Water, barley malt, hops and yeast | <10.0 | | |
| Labelled GF _38 | Craft | Ale | Water, barley malt, hops and yeast | <10.0 | | |
| Labelled GF _39 | Craft | Lager | * | <10.0 | | |
| Labelled GF _40 | Craft | Ale | Water, barley malt, hops and yeast | <10.0 | | |
| Labelled GF _41 | Industrial | Ale | Barley, oat, rye, buckwheat | <10.0 | | |
| Conventional _1 | Industrial | Ale | Barley malt, wheat malt and wheat | 60.6 | 15.4 | 164.2 |
| Conventional _2 | Industrial | Ale | Barley and wheat malt | 55.0 | 7.6 | 39.6 |
| Conventional _3 | Industrial | Lager | Barley malt | 32.0 | 1.6 | 12.7 |
| Conventional _4 | Craft | Ale | Water, barley malt, hops, yeast | 65.8 | 8.9 | 22.1 |
| Conventional _5 | Industrial | Lager | Water, barley malt, hop extract | 34.8 | 4.6 | 12.9 |
| Conventional _6 | Industrial | Lager | Water, barley malt, corn, hops | <10.0 | <0.25 | <3.6 |
| Conventional _7 | Industrial | Lager | Water, barley malt, corn, hops | 41.2 | 6.2 | 12.8 |
| Conventional _8 | Industrial | Lager | Water, barley malt, corn, rice, hops | <10.0 | <0.25 | <3.6 |
| Conventional _9 | Craft | Lager | Water, barley malt, hops, yeast | 37.0 | 7.3 | 18.3 |
| Conventional _10 | Industrial | Lager | Water, malta barley, corn, hops, yeast | 114.2 | 20.3 | 37.9 |
| Conventional _11 | Industrial | Lager | Water, barley malt, maltose, hop extract | 35.0 | 4.7 | 8.9 |
| Conventional _12 | Craft | Ale | Water, barley and wheat malt, hops, yeast | 100.8 | 26.8 | 107.2 |
| Conventional _13 | Craft | Ale | Water, barley malts, hops, yeast | 26.8 | 1.0 | 7.7 |
| Conventional _14 | Industrial | Lager | Water, barley malt, hops, hop extract | 63.4 | 9.5 | 19.0 |
| Conventional _15 | Industrial | Lager | Water, barley malt, corn, hop extract | 63.8 | 6.3 | 13.8 |

| Beer ID | Manufacturer | Yeast style | Ingredients | Gluten content mg/kg | | |
|---|---|---|---|---|---|---|
| | | | | R5 competitive | R5 direct | α20gliadin competitive |
| Conventional _16 | Industrial | Lager | Water, barley malt, corn, barley, hops | <10.0 | <0.25 | 4.4 |
| Conventional _17 | Industrial | Ale | Water, wheat malt, barley malt, hop extract | 1169.8 | 90.0 | 1327.2 |
| Conventional _18 | Industrial | Ale | Water, barley malt, glucose syrup, wheat malt, aromatic caramel, hops | 148.8 | 54.0 | 238.6 |
| Conventional _19 | Industrial | Ale | Water, wheat malt, barley malt, yeast, hops | 3716.6 | 503.8 | 1881.0 |
| Conventional _20 | Industrial | Lager | Water, barley malt, treated hops, hop extract | 43.4 | 5.9 | 25.6 |
| Conventional _21 | Industrial | Lager | Water, barley malt, corn, hops | 60.4 | 10.0 | 21.7 |
| Conventional _22 | Craft | Ale | Barley malta; wheat flakes, cande sugar, dextrose, hops, yeast | 160.8 | 49.9 | 74.6 |
| Conventional _23 | Craft | Ale | Barley malta; oat malt, hops, dextrose, yeast | 80 | 14.8 | 11.9 |
| Conventional _24 | Industrial | Lager | Barley malt | 25.2 | 2.5 | 5.0 |
| Conventional _25 | Industrial | Lager | Barley malt | 17.6 | 5.0 | 3.6 |
| Conventional _26 | Craft | Ale | Water, barley malt, hops, yeast | 67.8 | 19.1 | 26.7 |
| Conventional _27 | Industrial | Ale | Water, wheat and barley malt, hops, yeast | 1241 | 151.1 | 762.2 |
| Conventional _28 | Industrial | Ale | Water, barley malt, hops, yeast | 31.6 | 4.8 | 15.2 |
| Conventional _29 | Industrial | Lager | Water, 2 barley malts, 1 wheat malt, 4 varieties of hops, yeast | 248 | 108.7 | 439.2 |
| Conventional _30 | Industrial | Ale | Water, 3 barley malts, 1 wheat malt, 4 varieties of hops, yeast | 1036.8 | 78.0 | 757.2 |
| Conventional _31 | Industrial | Lager | Water, two barley malts, 1 wheat malt, 4 varieties of hops, yeast | 628.4 | 83.2 | 515.8 |
| Conventional _32 | Industrial | Lager | Water, barley malt, hops, hop extract | 26.8 | 4.3 | 19.0 |
| Conventional _33 | Industrial | Lager | Water, barley malt, rice, corn, hops | <10.0 | <0.25 | <3.6 |
| Conventional _34 | Craft | Ale | Water, barley malt, hops, yeast | <10.0 | <0.25 | 7.2 |
| Conventional _35 | Craft | Ale | Water, barley malt, hops, yeast | 23.6 | 2.2 | 7.0 |
| Conventional _36 | Industrial | Lager | Water, barley malt, hops, hop extract | 38.0 | 10.2 | 19.4 |
| Conventional _37 | Industrial | Ale | Barley and wheat malt | 2192.4 | 278.2 | 828.0 |
| Conventional _38 | Industrial | Ale | Water, wheat malt, barley malt, hop extract, yeast | 3171.4 | 434.4 | 1885.4 |
| Conventional _39 | Craft | Ale | Water, hops, yeast, barley malta | 40.8 | 9.2 | 11.6 |
| Conventional _40 | Craft | Ale | Water, hops, yeast, wheat malt, barley malt | 1363.2 | 166.4 | 1219.6 |
| Conventional _41 | Craft | Ale | Water, hops, yeast, barley malt | 111.4 | 14.0 | 33.0 |
| Conventional _42 | Industrial | Ale | Water, barley malt, sugar, hops | 51.8 | 16.3 | 23.5 |
| Conventional _43 | Industrial | Ale | Water, barley malt, sugar, spices (orange peel, coriander seeds), hops | 120.8 | 41.8 | 81.3 |
| Conventional _44 | Industrial | Lager | Water, barley malt, treated hops, hop extract | 23.8 | 5.4 | 18.3 |
| Conventional _45 | Craft | Ale | Water, barley malt, hops, yeast | 33.4 | 5.3 | 11.8 |

| Beer ID | Manufacturer | Yeast style | Ingredients | Gluten content mg/kg | | |
|---|---|---|---|---|---|---|
| | | | | R5 competitive | R5 direct | α20gliadin competitive |
| Conventional _46 | Industrial | Lager | Barley and barley malt | 61.8 | 8.9 | 32.2 |
| Conventional _47 | Craft | Ale | Water, barley malt, hops, yeast | 44.8 | 5.0 | 24.2 |
| Conventional _48 | Industrial | Ale | Water, barley malt, wheat malt, hops, yeast | 245.2 | 94.8 | 745.6 |
| Conventional _49 | Industrial | Ale | Barley, wheat and oatmeal. Orange peel, coriander | 2773.6 | 391.8 | 1558.0 |
| Conventional _50 | Craft | Ale | Barley malt | 65.8 | 11.3 | 39.0 |
| Conventional _51 | Industrial | Lager | Water, barley malt, corn, barley, hops, coloring E-150c, stabilizer E-405 | <10.0 | | |
| Conventional _52 | Industrial | Lager | Water, barley malt, rice, hops | <10.0 | | |
| Conventional _53 | Industrial | Ale | Water, barley and wheat malt, hops and yeast | 2039.6 | | |
| Conventional _54 | Craft | Lager | Water, barley malt, hops and yeast | <10.0 | | |
| Conventional _55 | Industrial | Lager | Water, barley malt, hops | <10.0 | | |
| Conventional _56 | Craft | Ale | Water, barley malt, hops and yeast | 31.0 | | |
| Conventional _57 | Industrial | Lager | Water, barley malt, corn, hops | 16.4 | | |
| Conventional _58 | Industrial | Ale | Water, wheat malt, barley malt, hops and yeast | 2754,2 | | |
| Conventional _59 | Industrial | Lager | Water, barley malt, rice, hops | < 10.0 | | |
| Conventional _60 | Craft | Ale | Water, barley malt, hops and yeast | 10.0 | | |
| Conventional _61 | Craft | Ale | Water, barley malt, hops and yeast | 40.2 | | |
| Conventional _62 | Craft | Ale | Water, barley malt, wheat malt, orange peel, coriander, hops and yeast | 234,4 | | |
| Conventional _63 | Industrial | Ale | Water, malt (Maris Otter), hops (Cascade and Simcoe), malted wheat and yeast (Ale) | < 10.0 | | |
| Conventional _64 | Industrial | Ale | Water, malt (Extra Pale), hops (Cascade and Mosaic) and yeast (Ale) | < 10.0 | | |
| Conventional _65 | Craft | Ale | Water, malt, hops, yeast, caramel | 62.4 | | |

GF: gluten free. *The manufacturer does not provide raw materials on the label.
